# Building an eDNA surveillance toolkit for invasive rodents on islands: can we detect wild-type and gene drive *Mus musculus*?

**DOI:** 10.1186/s12915-024-02063-0

**Published:** 2024-11-15

**Authors:** Antoinette J. Piaggio, Luke Gierus, Daniel R. Taylor, Nick D. Holmes, David J. Will, Neil J. Gemmell, Paul Q. Thomas

**Affiliations:** 1 U.S. Department of Agriculture, Animal Plant Health Inspection Service, Wildlife Services, National Wildlife Research Center, Fort Collins, CO USA; 2 School of Biomedicine and Robinson Research Institute, University of Adelaide, Adelaide, Australia; 3 Genome Editing Program, South Australian Health and Medical Research Institute, Adelaide, Australia; 4 The Nature Conservancy, Santa Cruz, CA USA; 5 Island Conservation, Santa Cruz, CA USA; 6 Department of Anatomy, School of Biomedical Sciences, University of Otago, Dunedin, New Zealand

**Keywords:** Environmental DNA (eDNA), *Mus musculus*, CRISPR/Cas, Gene drive, Invasive species

## Abstract

**Background:**

Invasive management strategies range from preventing new invasive species incursions to eliminating established populations, with all requiring effective monitoring to guide action. The use of DNA sampled from the environment (eDNA) is one such tool that provides the ability to surveille and monitor target invasive species through passive sampling. Technology being developed to eliminate invasive species includes genetic biocontrol in the form of gene drive. This approach would drive a trait through a population and could be used to eliminate or modify a target population. Once a gene drive organism is released into a population then monitoring changes in density of the target species and the spread of the drive in the population would be critical.

**Results:**

In this paper, we use invasive *Mus musculus* as a model for development of an eDNA assay that detects wild-type *M. musculus* and gene drive *M. musculus*. We demonstrate successful development of an assay where environmental samples could be used to detect wild-type invasive *M. musculus* and the relative density of wild-type to gene drive *M. musculus*.

**Conclusions:**

The development of a method that detects both wild-type *M. musculus* and a gene drive *M. musculus* (*t*_CRISPR_) from environmental samples expands the utility of environmental DNA. This method provides a tool that can immediately be deployed for invasive wild *M. musculus* management across the world. This is a proof-of-concept that a genetic biocontrol construct could be monitored using environmental samples.

**Supplementary Information:**

The online version contains supplementary material available at 10.1186/s12915-024-02063-0.

## Background

Effective detection of invasive species is a critical need in efforts to reduce or halt the damage they cause to native biodiversity. Invasive rodents are among the world’s most harmful invasive species given known impacts to native species and ecosystems, human health, food security, and economic activity [1–4]. Impacts have proven particularly acute for native biota and ecosystem functioning on islands [5, 6], where species often evolved in the absence of mammalian predators [7], and there are disproportionately higher rates of endemism by area compared to mainlands [8, 9]. Invasive rats (*Rattus rattus*, *R. norvegicus*) and mice (*Mus musculus*) are widespread on islands—80% of the world’s archipelagos are estimated to be invaded by rats [10]—arriving as unintentional hitchhikers on vessels and other transport. As generalists, these invasive rodents predate a wide variety of plants, seeds, invertebrates, and vertebrate taxa leading to reduced native island species abundances, extirpations, extinctions, and negative impacts on ecosystem system function on land and in nearshore environments [5, 10–17]. Accordingly, management strategies to mitigate harmful invasive rodent impacts are high priority for islands [18].

Two core strategies for mitigating harmful impacts from invasive rodents to islands are biosecurity, to prevent new populations becoming established, and eradication, to free part or entire islands from these species altogether [18–21]. Biosecurity consists of preventing, detecting, and rapidly responding to new invasive species incursions and is more cost-effective and tractable than eradication of extant populations [22–24]. Eradication of invasive populations of rodents has proven to be successful for more than 500 islands [25], with demonstrable benefits for biodiversity [26, 27]. However, eradication of rodent populations from islands relies on toxicants that pose an added environmental burden, animal welfare concerns, and off-target effects [11]. Surveillance to detect rodents at low density plays a key role in both strategies, either to trigger a rapid response to an incursion before a breeding population can be established or informing the success or failure of an eradication attempt.

Although current eradication methods have been successful, the development of new tools is critical as toxicants are not feasible for all use cases and ecological damage from invasive rodents is an intractable problem. A future potential eradication strategy that could mitigate the challenges posed by current methods is genetic biocontrol. Such an approach could minimize unwanted effects from current toxicant-based tools and overcome barriers to currently intractable eradication targets [28–30]. Genetic biocontrol would entail engineering a gene drive to bias transmission of a genetic trait (e.g., sex ratio) in an invasive population to drive it to zero [31, 32]. Foundational lab-based research is underway, including development of a gene drive strategy that targets female fertility in *M. musculus* [33] and could ultimately be designed to be population-specific [34, 35]. Future field-based testing using oceanic islands and invasive *M. musculus* offers unique learning opportunities and a naturally biosecure experiment [29, 32, 36]. Surveillance will play a critical role in evaluating future field testing. It will be necessary to have the ability to monitor the spread of the gene drive in the wild invasive *M. musculus* population to evaluate efficacy, monitor the expected wild-type decline, and ensure genetic and physical containment mechanisms are maintained. Ideally, such surveillance would not require invasive sampling of individual *M. musculus* to eliminate biasing outcomes of testing gene drive transfer though the wild population.

Current methods of surveillance include detection dogs, remote cameras, tracking tunnels, chew cards, and trapping [18, 37, 38]. However, genomics has played an increasingly prevalent role in informing and designing biosecurity strategies, eradication programs, and risk assessments [38–40]. Sampling of environmental DNA (eDNA), which is found in water, air, soil, and on surfaces, offers an approach for surveillance and has been found to be a useful addition to this suite of monitoring tools (e.g., [38, 39, 41–44]). Application of eDNA will hold promise for evaluating the efficacy of any genetic biocontrol of rodents. Many eDNA studies have focused on detecting species presence and absence or for biodiversity assessments [45]; however, there is an emerging research area investigating the use of eDNA to detect population-level genetic information [46], intra-species genetic variation [47], and infer environmental change at landscape levels [48]. These approaches would allow for in-depth passive monitoring of impacts of biocontrol to targeted invasive species and the native biodiversity as well.

**Fig. 1:**
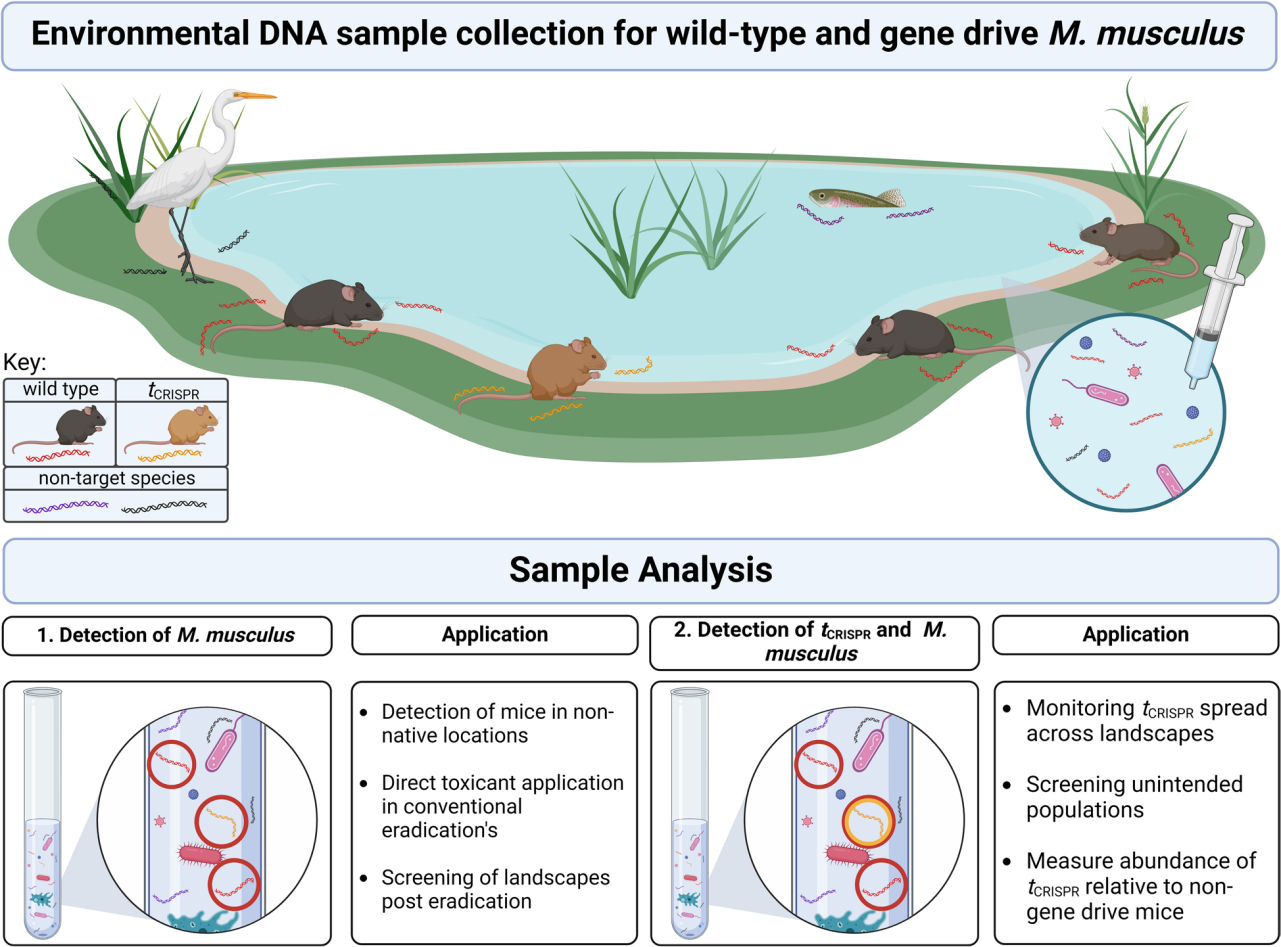
A graphical representation of DNA being shed by different species, including *M. musculus* (red for wild-type (WT), yellow for tCRISPR mice), a bird (black DNA molecule), and a fish (purple DNA molecule), into the environment. DNA molecules are then collected by water sampling, isolated in the laboratory, and target DNA amplified through quantitative polymerase chain reaction (qPCR). In this scenario, gene drive tCRISPR *M. musculus* have been released to interbreed with WT mice and suppress the population. Our assays have been designed to detect the shed DNA from [1] all *M. musculus* (circled in red), including the tCRISPR *M. musculus*, and [2] tCRISPR *M. musculus* specifically (circled in yellow) and can be multiplexed with assay [1]. This multiplexed assay allows detection of tCRISPR DNA relative to WT *M. musculus*, enabling monitoring of relative abundance and spread of tCRISPR gene drive in the population. DNA from other species may be collected but do not amplify in our species-specific assay. Figure 1 created using BioRender https://app.biorender.com

In this study, we developed a multiplex of two eDNA assays for detecting *M. musculus* to aid invasive species mitigation strategies on islands and provide a model for future research assays on other invasives, such as rats. We have developed a *M. musculus* eDNA quantitative PCR (qPCR) assay for the detection of *M. musculus* from environmental samples to aid island biosecurity and post-eradication monitoring. The second eDNA assay, which can be multiplexed with the first, detects a proposed murine meiotic gene drive—*t*_CRISPR_ [33]. When both assays are combined, the relative increase in *t*_CRISPR_ gene drive compared to wild type can be monitored; thus, the environment can be sampled for presence or absence of both and relative abundance of each without need for trapping efforts or as complement to trapping efforts (Fig. 1). This marks a novel use of eDNA to detect the presence and relative abundance of the gene drive organism, with implications for improved surveillance for the broader field of gene drive research and applications of synthetic biology in invasive species management. Furthermore, the use of eDNA in terrestrial systems is less advanced than marine systems, and there are very few studies that demonstrate the ability to monitor specific genetic traits in the environment. By demonstrating that eDNA assays can reliably detect specific genetic traits in *M. musculus*, we intend to build evidence for the development of future non-invasive genetic surveillance tools capable of informing risk assessment and decision-making over large spatial and temporal scales that today’s tools cannot.

## Methods

### *Mus musculus* wild-type (WT) assay

We designed our *M. musculus* wild-type (WT) eDNA qPCR assay (targeting the mitochondrial DNA cytochrome c oxidase subunit I (COI) gene with a forward primer (5′-CCAATCACAAAGATATCGGAACC-3′), reverse primer (5′-CTGGTTGACCTAATTCTGCTCG-3′), and probe (5′-/5HEX/CCTGAGC[+ G]GGAATAGT[+ G]GG[+ T]A/3IABkFQ/-3′). We designed this WT assay with a probe containing three locking nucleotides placed with the intent to multiplex with different probes that together could distinguish between three subspecies of *M. musculus*: *M. m. domesticus*,* M. m. musculus*, and *M. m. castaneus*. While this subspecies multiplex assay did not successfully distinguish each subspecies due to cross amplification in preliminary testing, the locking nucleotides were kept due to the success of the *M. m. domesticus* assay at amplifying each of the three subspecies. The first locking guanine towards the 5′ end of the probe was added to reduce potential for non-target binding with *Rattus* species, as *Rattus* have an adenine in that position. Primers were designed with the help of PrimerQuest™ program, Integrated DNA Technologies (IDT) (Coralville, Iowa, USA), OligoAnalyzer™ (IDT), and the National Center for Biotechnology Information (NCBI) GenBank database. Parameters were set such that the amplicon had to be less than 150 bp, annealing temperatures 60 °C–67 °C, length of primers 20 bp–27 bp, and other parameters were not changed. We designed and ordered synthetic gBlocks® Gene Fragments from IDT to use as standards for our WT target (5′-CTCAACCAATCACAAAGATATCGGAACCCTCTATCTACTATTCGGAGCCTGAGCGGGAATAGTGGGTACTGCACTAAGTATTTTAATTCGAGCAGAATTAGGTCAACCAGGTGCACTTTTAGGAG-3′). Standards for the assay were run in triplicate at 5 × 10^5^, 5 × 10^4^, 5 × 10^3^, 5 × 10^2^, and 5 × 10^1^ copies/rxn. We optimized the WT assay conditions for best performance using our gBlocks® standards and genomic DNA collected from tissue of *M. musculus.* Using a gradient for assessing optimized thermocycling conditions on a CFX96 C1000 Touch Real-Time PCR Detection System (Bio-Rad Laboratories, Hercules, CA, USA) found that 95 °C 10 min followed by (95 °C 15 s, 60 °C 60 s) × 50 cycles achieved the best results. Amplifications were optimized as a 30-μL reaction composed of 15 μL of 2 × TaqMan Environmental Mastermix 2.0 (Life Technologies, Carlsbad, CA, USA), 1 μL of each 10 μM primer, 1 μL of 2.5 μM probe, 1 μL of 5 mg/ml bovine serum albumin, 1 μL of 100% dimethyl sulfoxide, 5 μL of laboratory-grade sterile water, and 5 μL of template.

### Species-specificity

We performed in silico analyses to ensure our chosen primers and probe did not cross-amplify non-target taxa. First, we used eDNAssay, a machine learning tool to assess primer/probe specificity [49]. We set the forward primer melting temperature (T_m_) at 62, reverse T_m_ at 64, and the probe T_m_ at 70. We tested primer specificity against many *M. musculus* subspecies (*M. m. musculus*, *M. m. castaneus*, and *M. m. domesticus*) sequences, *Rattus* spp., and other common species (input file at https://zenodo.org/records/12609243). Next, we used nucleotide BLAST (https://blast.ncbi.nlm.nih.gov/Blast.cgi) on each primer and our probe independently and excluded our target taxa, *Mus musculus*, to assess percent identity to other species, including closely related species. We set the BLAST analysis to return a maximum 5000 off-target sequences. Using gBlock (above), we also used the nucleotide BLAST feature of NCBI optimizing for highly similar sequences limited to 250 returned significant alignments. Finally, we tested specificity of the WT assay in vitro by testing for cross-species amplification of genomic DNA isolates of other vertebrate species that were available in our laboratory. These DNA isolates included five species of rats and 16 other vertebrate species (Supplementary Info 1) with the goal of testing species that might be found in proximity to mice commonly or a broad taxonomic range of species to assess species specificity of the primers.

### Limit of detection/quantitation

We evaluated WT assay performance following a limit of detection (LOD) and limit of quantification (LOQ) analysis as described in Klymus et al. [50] and Mangan et al. [51]. Briefly, we define LOD as the lowest concentration of target DNA (copies/μL) that can be amplified with a 95% detection rate, and LOQ is the lowest concentration that can be quantitatively determined within a stated precision (we used a coefficient of variation [CV] of 35%; 51). We selected the option “Best” to allow the R code (10.5281/zenodo.12609243) to select the best fitting model choice for LOD and the model with the lowest residual standard error for LOQ [50–52]. Three independent qPCR plates were analyzed, each with 16 replicates of five dilution levels of gBlocks® standards (*n* = 48 each at 500, 50, 5, 0.5, 0.05 copies/rxn) in 1XTE buffer (10 mM Tris–HCl (pH 8.0) 0.1 mM EDTA, Thermo Fisher Scientific, Waltham, MA, USA) on a CFX96 C1000 Touch Real-Time PCR Detection System (BioRad, USA). Forty-eight blanks (no template controls) were also included to monitor primer-dimer formation, laboratory contamination, and other false positives (10.5281/zenodo.12609243).

### *t*_*CRISPR*_* primer design*

The *t*_CRISPR_*M. musculus* was generated according to Gierus et al. [33]. In brief, the *t*_CRISPR_ is a transheterozygous *M. musculus* containing the Tg(Ccna1-cas9,-EGFP)1Pqt transgene (MGI:7,294,475) and the *t*^em1(U6−gRNA:Prl,CMV−mCherry)Pqt^ transgene (MGI:7,294,476). For the purposes of this assay, the *t*^em1(U6−gRNA:Prl,CMV−mCherry)Pqt^ is the relevant transgene, integrated within an intergenic region in inversion 1 of the *t* haplotype. This transgene is composed of a CMV-mCherry cassette in the forward direction from pZ148-BhCas12b-sgRNA-scaffold (Addgene plasmid #122,448) and U6-gRNA:Prl-HuU6-3′ in the reverse direction generated via gBlock® synthesis (IDT) (Table 1). The *t*_CRISPR_ eDNA primers/probe were designed where the forward primer (5′-CTCTCTAACAGCCTTGTATCGT-3′) was designed to bind to the CMV-mCherry cassette, the probe (5′-56-FAM/AAGGAATCA/ZEN/TGGGAAATAGGCCCTCC/3IABkFQ/-3′) sits on the vector sequence, and the reverse primer (5′-CTTTGATGGGCAGAGCAATC-3′) sits on the *M. musculus* chromosome 17. Primers were designed with the help of PrimerQuest™ program (IDT) and (NCBI) GenBank database with the same parameters as our WT assay. We designed and ordered synthetic gBlocks® Gene Fragments from IDT to use as standards for our *t*_CRISPR_*M. musculus* target (5′-CTCTCTAACAGCCTTGTATCGTATATGCAAATATGAAGGAATCATGGGAAATAGGCCCTCCCCAGGTACATGTCTGTTCCTATTTCCTGCAGAGATTGCTCTGCCCATCAAAGGATGGCACAGCC-3′).

**Table 1:**
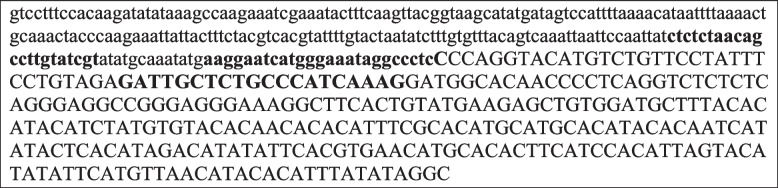
Portion of the gene drive construct for *t*_CRISPR_ *M. musculus* primers and probes are in bold (5’-3’)

The forward primer sits in the hU6 promoter in the *t*_CRISPR_ transgene, the probe spans the transgene, and *t *haplotype junction, and the reverse primer sits in the *t *haplotype

lowercase letters, hU6 promoter

Capital letters, chromosome 17 tw2 sequence / Right homology arm

### Relative abundance test

We tested our *t*_CRISPR_ assay for specificity and performance relative to our WT assay by performing both assays separately under the same conditions as described above with various template controls. Template controls in these tests included DNA extracted from the tissues of both wild-type and *t*_CRISPR_ DNA as well as diluted and mixed ratios of both (Table 2). Mixed genomic DNA samples of both were quantified using a Qubit 2.0 fluorometer (Thermo Fisher Scientific) and diluted to a starting concentration of 5 ng/μL before seral dilutions and mixing.

**Table 2:** Relative abundance test results for qPCR assays that target DNA from either *Mus musculus* cytochrome c oxidase subunit I (COI) gene or* t*_CRISPR_ gene-edited *M. musculus*

Ratio WT *M. musculus*:* t*_CRISPR_*M. musculus*	*M. musculus* WT assay Cq^a^	*t*_CRISPR_*M. musculus* assay Cq^a^
1:0	18.39	No amplification
0:1	17.78	24.76
1:1	17.02	24.63
0.1:1	17.73	24.26
0.01:1	17.71	24.35
0.001:1	17.83	24.29
0.0001:1	17.84	24.49
1:0.1	18.14	28.32
1:0.01	18.24	32.14
1:0.001	18.14	37.09
1:0.0001	18.22	39.90

^a^*Cq*, cycle quantification

Genomic DNA from a wild-type (WT) *M. musculus* and a* t*_CRISPR_*M. musculus* was diluted to 5 ng/ μL, serial diluted to the corresponding ratio, then combined. *M. musculus* WT assay was designed to amplify *M. musculus*; thus, Cq values are not significantly changed when* t*_CRISPR_ gene-edited *M. musculus* is also present in the sample. The *t*_CRISPR_*M. musculus* assay was designed to amplify only* t*_CRISPR_ gene-edited *M. musculus.* Thus, Cq values are not changed as the amount of wild-type *M. musculus* DNA changes, while Cq values increase as* t*_CRISPR_*M. musculus* DNA is diluted. Quantitative PCR efficiencies are shown in Supplementary Info 1

### Multiplex assay

Assay conditions for the multiplex were the same cycling parameters and reagents as the singleplex WT assay except with 2 μL of water instead of 5 μL to accommodate for the extra set of primers/probes. The multiplex assay was made from separate serial dilutions of both *M. musculus* WT and *t*_CRISPR_ gBlocks® standards then combined at a 1:1 ratio and run in triplicate at 5 × 10^5^, 5 × 10^4^, 5 × 10^3^, 5 × 10^2^, and 5 × 10^1^ copies/rxn.

### Multiplex limit of detection/quantitation

We evaluated our multiplex assay performance following an LOD/LOQ analysis as described above and following Klymus et al. [50] and Mangan et al. [51] but with 1:1 ratio gBlocks® standards of 2500, 250, 25, 2.5, and 0.25 copies/rxn and 39 no-template controls (10.5281/zenodo.12609243). We also included three positive controls of genomic DNA isolated from WT *M. musculus*,* t*_CRISPR_*M. musculus*, and a 1:1 ratio of both to the 3 LOD/LOQ plates to further test the multiplex reaction on target DNA.

## Results

### *Mus musculus* WT assay performance and species-specificity

The *M. musculus* wild-type (WT) assay was optimized using gBlock standards and genomic DNA from *M. musculus*. Specificity to the *M. musculus* target was high as determined initially through a Primer-BLAST, eDNAssay, and a search of both primers and probe on the NCBI GenBank database. Testing for primer specificity using eDNAssay yielded results showing that amplification predictions for *M. musculus* subspecies ranged from 0.82 to 0.86, and the next best amplification prediction was for *R. norvegicus* at 0.42 (10.5281/zenodo.12609243). NCBI GenBank database found that *R. norvegicus* has 100% query coverage and 91%, 82%, and 86% percent identity match with the forward primer, reverse primer, and probe, respectively. We further used a BLAST search on the gBlock sequence, including primer sequence, while excluding *M. musculus* from the search. There was a single *M. spretus* match of 100% query coverage/100% identity, and all other matches were *Mus* spp. with 95–100% query coverage/94.02–95.83% identity. We then blocked all *Mus* spp. and the closest match was to *Mastomys* spp. with 94% query coverage/90.52% identity. Our in vitro cross-species amplification qPCR did not produce any amplification for any species, including *R. norvegicus*, other than *M. musculus* positive controls (Supplementary Info 1). Both in silico analyses and in vitro testing demonstrate high species specificity of the WT assay to *M. musculus* DNA in the absence of *M. spretus*.

### *Mus musculus* WT limit of detection/quantitation

**Fig. 2:**
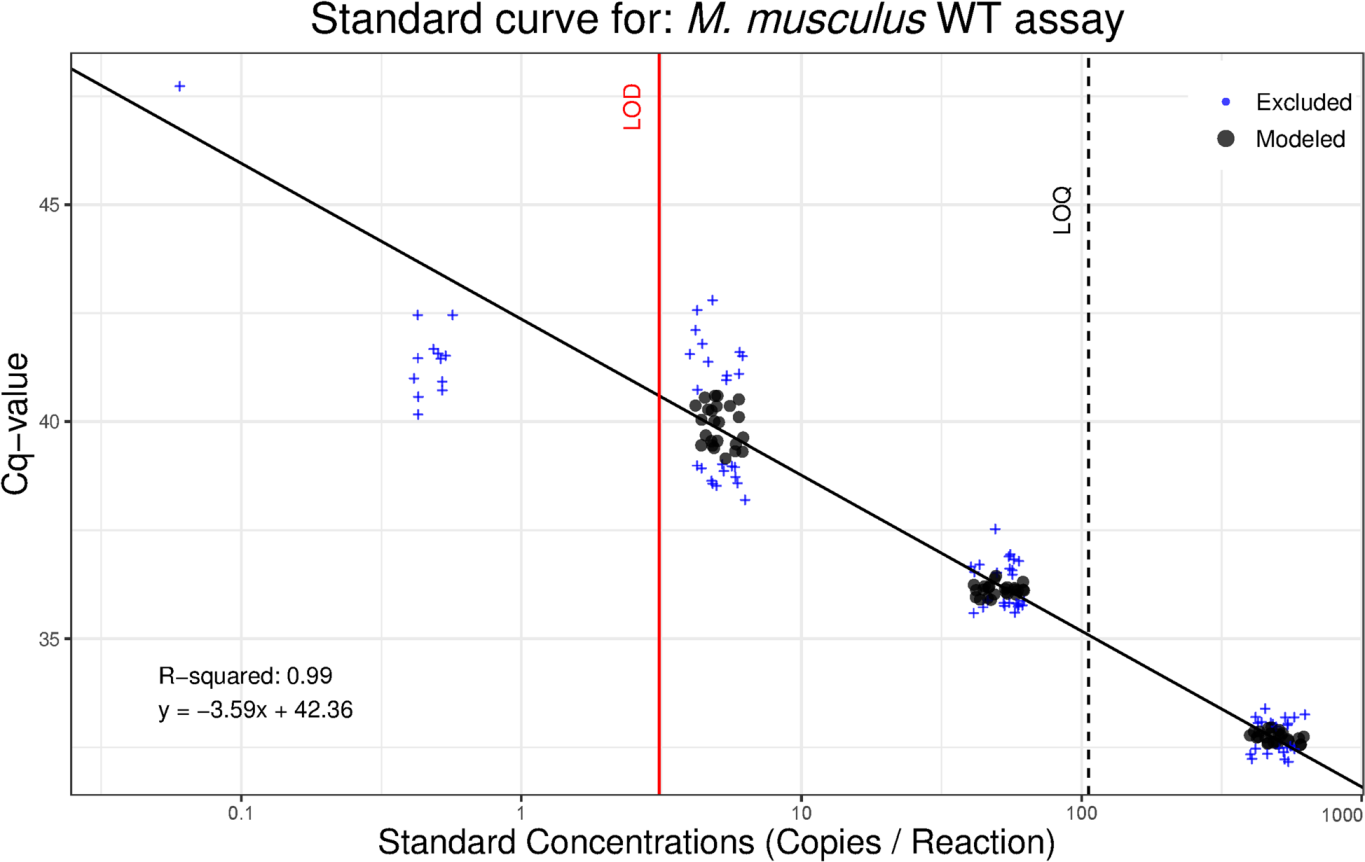
Limit of detection (LOD)/limit of quantification (LOD) analysis results for *Mus musculus* wild-type (WT) eDNA qPCR assay. Synthetic gBlocks® standards of the target DNA fragment were run in 48 replicates at five concentrations and modeled to determine the lowest concentration of target DNA that can be amplified with 95% detection and the lowest concentration that can be quantitatively determined within a coefficient of variation [CV] of 35%. Data points in black circles are the middle two quartiles of standards with ≥ 50% detection and are included in linear regression calculations. Data points in blue ( +) are outside the middle two quartiles or standards with < 50% detection and therefore not included in linear regression calculations

**Fig. 3:**
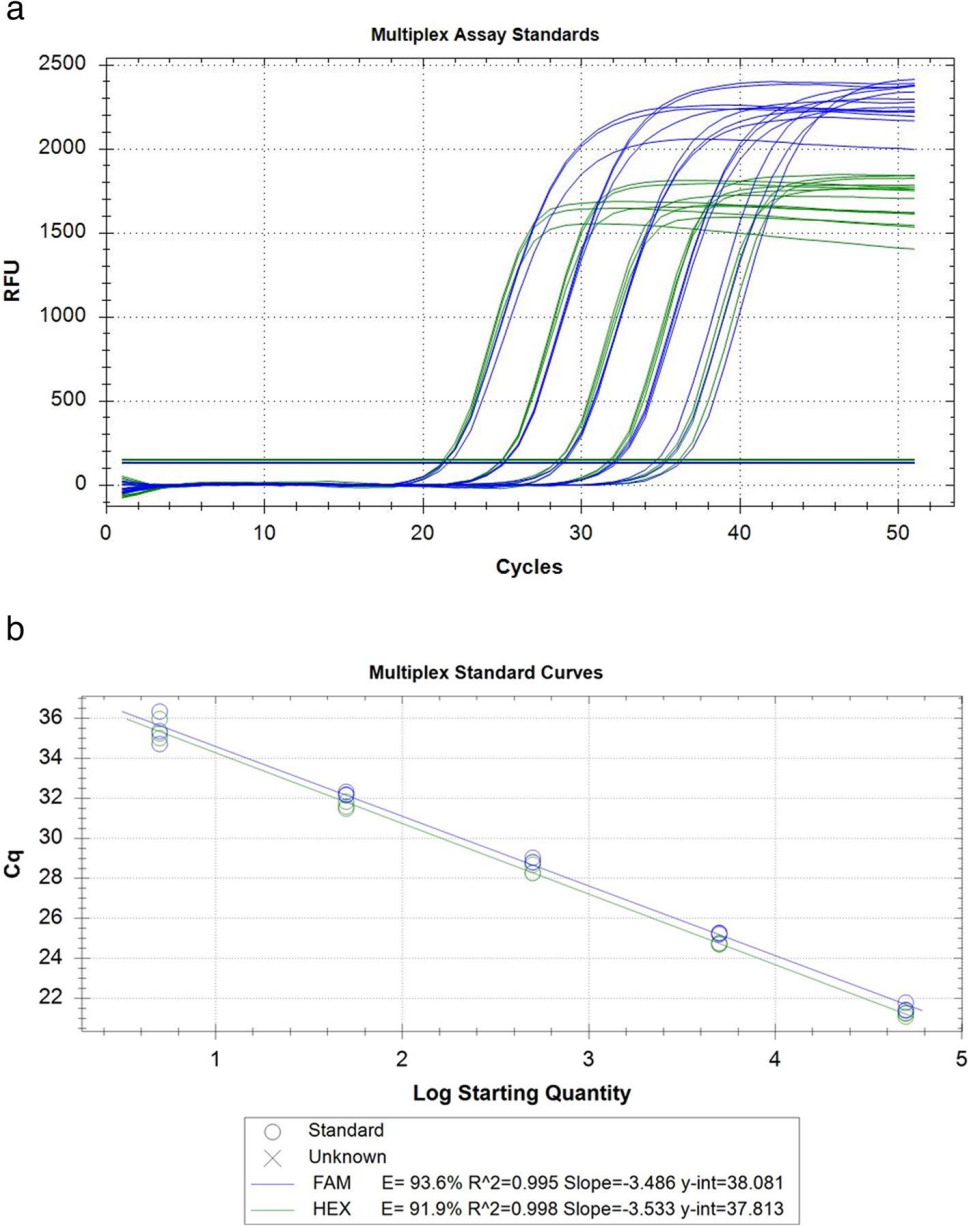
qPCR amplification (**A**) and standard curves (**B**) of gBlocks® standards in a multiplex assay where standards are made from separate serial dilutions of both wild-type (WT) and *t*_CRISPR_*Mus musculus* DNA then combined at a 1:1 ratio and run in triplicate at 5 × 10^5^, 5 × 10^4^, 5 × 10^3^, 5 × 10^2^, and 5 × 10^1^ copies/rxn. When WT (HEX) and *t*_CRISPR_ (FAM) *M. musculus* assays are multiplexed together, Cq values between the two amplified products are close together, showing that DNA from* t*_CRISPR_ gene-edited *M. musculus* will be expected to amplify with similar efficiency as WT *M. musculus* DNA

The best fitting model (probit model) of the LOD analysis determined the 95% LOD was 3.1 DNA copies/rxn, and the LOQ as determined by the best fit decay model (linear) was 106 copies/rxn (Fig. 2). Non-template controls had no amplification, suggesting an absence of primer-dimers, laboratory contamination, and no false positives (10.5281/zenodo.12609243). Efficiencies and linear dynamic ranges for each qPCR were high, but it is important to note that with LOD/LOQ you aim to have a dilution that is below the threshold to determine reliable detection limits but this leads to having standards that are below the limit of linearity (see Fig. 3 Klymus et al. 2020; Supplementary Info 1).

### Relative abundance test

The WT assay amplifies and detects both the WT and *t*_CRISPR_*M. musculus* DNA, which was confirmed by similar amplification of the two quantified genomic DNA extracts (wild-type *M. musculus* Cq = 18.39, *t*_CRISPR_*M. musculus* Cq = 17.78) as well as positive amplification of unquantified* t*_CRISPR_ (3/3; Cq range 15.49–18.19) and WT (3/3; Cq range 16.22–30.01) *M. musculus* DNA. Regardless of the ratio and presence of DNA from both types of *M. musculus* in samples (Table 2), amplification remains the same for both types using the WT assay (Cq values range 17.02–18.39). Our *t*_CRISPR_ assay was designed to amplify only *t*_CRISPR_*M. musculus* DNA, and this was confirmed by (3/3) amplification of *t*_CRISPR_*M. musculus* DNA and zero WT *M. musculus* DNA amplifications (0/3). Further testing of the *t*_CRISPR_ assay reveals that when samples contain a mix of WT and *t*_CRISPR_*M. musculus* DNA, amplification is not influenced by the amount of WT *M. musculus* DNA in the sample (Cq range 24.26–24.76; Table 2), while amplification of *t*_CRISPR_*M. musculus* DNA is reduced as *t*_CRISPR_*M. musculus* DNA is diluted (Cq increases to 39.9 at 10,000-fold dilution; Table 2). Thus, we can assume the only DNA being amplified by the *t*_CRISPR_ assay is *t*_CRISPR_*M. musculus* DNA. Efficiencies and linear dynamic ranges for each qPCR were robust (10.5281/zenodo.12609243).

### Multiplex assay

When our WT and *t*_CRISPR_*M. musculus* assays are multiplexed together, Cq values between the two amplified products are similar (Fig. 3), showing that DNA from* t*_CRISPR_ gene-edited *M. musculus* will be expected to amplify comparably as WT *M. musculus* DNA. Different maximum RFUs (relative fluorescence units) are expected due to HEX and 6-FAM dye chemistry (IDT). Both standard curves passed (> 90% efficiency and > 99% *r*^2^; Fig. 3; Supplementary Info 1).

### Multiplex limit of detection/quantitation

**Fig. 4:**
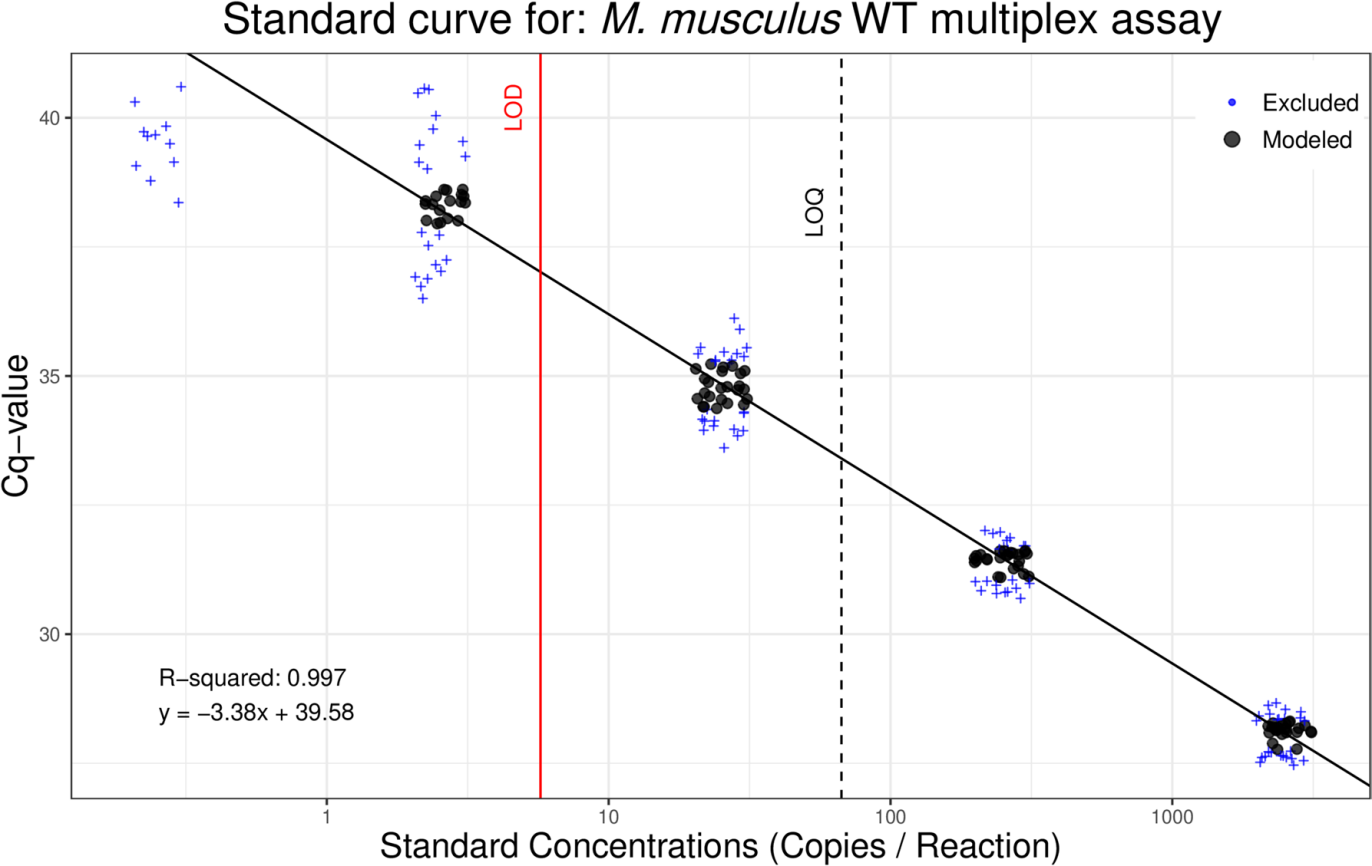
Limit of detection (LOD)/limit of quantification (LOD) analysis results for wild-type (WT) *M. musculus* when multiplexed in a qPCR assay that is also detecting *t*_CRISPR_*M. musculus* DNA. Synthetic gBlocks® standards of the target DNA fragment were run in 48 replicates at five concentrations and modeled to determine the lowest concentration of target DNA that can be amplified with 95% detection and the lowest concentration that can be quantitatively determined within a coefficient of variation [CV] of 35%. Data points in black circles are the middle two quartiles of standards with ≥ 50% detection and are included in linear regression calculations. Data points in blue ( +) are outside the middle two quartiles or standards with < 50% detection and therefore not included in linear regression calculations

**Fig. 5:**
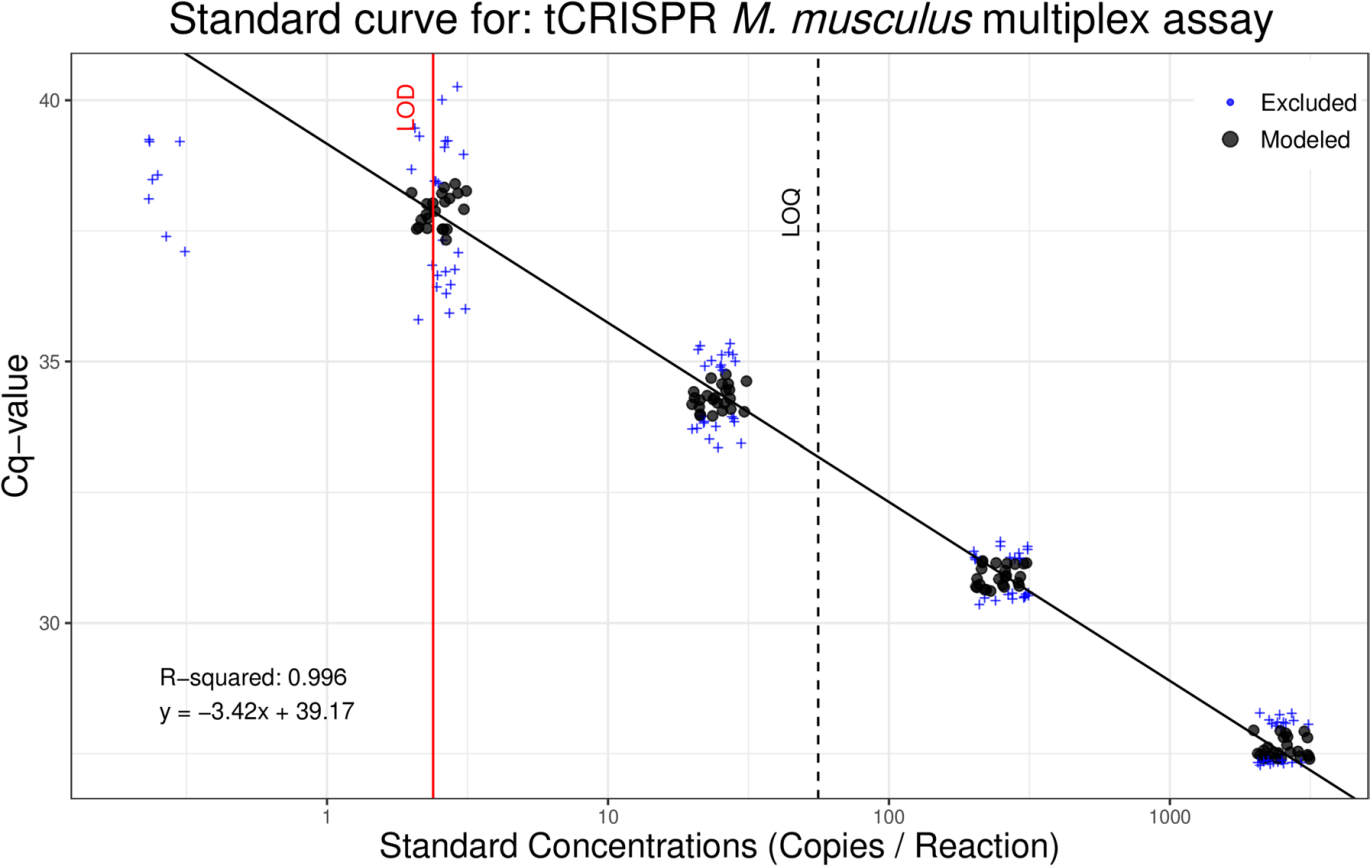
Limit of detection (LOD)/limit of quantification (LOD) analysis results for *t*_CRISPR_*Mus musculus* when multiplexed in a qPCR assay that is also detecting wild-type (WT) *M.s musculus* DNA. Synthetic gBlocks® standards of the target DNA fragment were run in 48 replicates at five concentrations and modeled to determine the lowest concentration of target DNA that can be amplified with 95% detection and the lowest concentration that can be quantitatively determined within a coefficient of variation [CV] of 35%. Data points in black circles are the middle two quartiles of standards with ≥ 50% detection and are included in linear regression calculations. Data points in blue ( +) are outside the middle two quartiles or standards with < 50% detection and therefore not included in linear regression calculations

The best fit models for the multiplex LOD/LOQ analysis determined WT assay LOD is 5.7 DNA copies/rxn, and LOQ is 67 copies/rxn (Fig. 4), while *t*_CRISPR_ assay LOD is 2.4 DNA copies/rxn, and LOQ is 56 copies/rxn (Fig. 5). The best fitting models for both assays were the probit model for LOD and 3-order polynomial exponential decay for LOQ. None of the no-template control replicates had amplification, suggesting an absence of primer-dimers, laboratory contamination, and other false positives (10.5281/zenodo.12609243).

Positive controls amplified as expected, where WT *M. musculus* DNA amplified each time with WT assay primers/probe, and there was no amplification with the *t*_CRISPR_ assay primers/probe. The *t*_CRISPR_*M. musculus* genomic DNA amplified with both sets of primers/probes.

Additionally, the combined template of both WT and *t*_CRISPR_*M. musculus* DNA amplified without significantly changing Cq values, suggesting there is no cross-reactivity between primers and probes when both assays are multiplexed or when DNA from both WT and *t*_CRISPR_*M. musculus* is present in the sample (WT assay MEAN Cq 20.80, standard deviation (Stdev) 0.28; *t*_CRISPR_ assay MEAN Cq 27.87, Stdev 0.21; Supplementary Info 1).

## Discussion

The developed eDNA WT *M. musculus* assay has utility for many applications. At the most basic application, it can be used to assess the presence of wild *M. musculus* from environmental samples (water, soil, air, swabs of plants, etc.). This approach, paired with other monitoring methods [38], would be a powerful layered detection tool for biosecurity to understand if *M. musculus* have invaded an island or for monitoring after an *M. musculus* eradication to evaluate success or failure. We also anticipate utility evaluating if islands are free of *M. musculus* after *Rattus* spp. eradication, given eradication of *Rattus* spp. has sometimes resulted in previously undetected small populations of *M. musculus* expanding once being released from these larger, more competitive congeners [53]. The most advanced application of the assay reported in this study would be to multiplex both primer sets to detect the presence/absence of both WT and gene drive *M. musculus* and, if sampled over time, to track the change in abundance of *M. musculus* with a heritable gene drive versus WT individuals. The success or failure of a gene drive release into a *M. musculus* population could thus be assessed without the need to conduct invasive monitoring of the population. We have demonstrated that these two sets of primers and probes, as a multiplex assay, can be run simultaneously with no evidence of loss of performance or primer/probe cross reactivity.

The development of the WT and *t*_CRISPR_*M. musculus* eDNA assays in a laboratory is the first step towards an environmental detection tool. The WT assay holds promise for assisting in current management efforts of invasive *M. musculus*. The next steps would be to assess efficacy in a mesocosm with a known number of animals and time point sampling before, during, and after animals are present. This would provide an understanding of how quickly *M. musculus* DNA accumulates once they are introduced and how long it persists once they are removed. This is critical information to understanding when to start sampling, for example, after an eradication effort. Furthermore, there are standardized validation steps to achieve readiness for deployment in the field [54]. These steps include field testing and occupancy modeling to derive the best sampling strategy and effort for detection when numbers of individuals are low [54–56]. The prospect of use of the *t*_CRISPR_ assay is less clear, given the challenging path through gene drive development [33, 57], field trials [36], social, cultural [58–61], and regulatory evaluations [62, 63] that the gene drive *M musculus* would have to be assessed. However, this proof-of-concept demonstrates the potential for application of eDNA for monitoring of genomic biocontrol.

These assays can be easily adapted to droplet digital PCR (ddPCR) and allow for more precise quantification of copies/μl, possibly better sensitivity in the presence of inhibitors, and greater throughput capability, as space on the plate will not be taken up by standard curve [56, 64]. For each new application, there will need to be validation that the WT assay does not have any cross-reactivity with unique species present in the target ecosystem and LOD/LOQ assessed in each lab the assay is performed [50]. In this study, we tested cross-reactivity across various taxonomic groups, but we cannot be inclusive of all possibilities, especially on islands where there are many endemic species. To realize the greater value of our approach for invasive species management requires further research in laboratory and natural settings to understand *M. musculus* DNA persistence, which informs probabilities of detection of low quantity/quality DNA in the target environment and the development of robust sampling strategies necessary to apply this tool in a management context. Finally, when applying the WT *M. musculus* assay, it would be critical to further assess cross-species amplification with co-occurring species for the target application area. We could not test all possible species given *M. musculus* world-wide distribution, particularly on islands that have endemic species that were not evaluated with the designed primers.

## Conclusions

This proof-of-concept that eDNA could be harnessed to detect a *M. musculus* (*t*_CRISPR_) gene drive laboratory construct also means that eDNA assays can be used to detect and monitor other genetic traits of interest, such as genetic resistance to anticoagulant rodenticide, presence of naturally occurring *t*-haplotypes that would prohibit the release of *t*_CRISPR_ on an island, or allelic variants that are associated with a particular disease status [47]. Multiplexed eDNA assays have great potential for the fields of gene drive research, synthetic biology, and biocontrol as stakeholders seek to evaluate, assess, and apply next generation methods. The current means for assessing the efficacy and spread of existing and future biocontrol methods (e.g., gene drive, transgenic release, SIT) require destructive sampling from individuals, which may affect efficacy of the biocontrol in the wild population. eDNA offers a population level assessment of genetic traits of interest that could allow stakeholders to monitor the relative efficacy of different methods at watershed or management zone scales, enabling management decisions on scales not currently possible.

## Supplementary Information

Supplementary Material 1: Species-specificity and qPCR efficiency.

## Data Availability

The datasets generated and/or analysed during the current study are available in supplementary information and the Zenodo repository, 10.5281/zenodo.12609243.
